# Supplementary material for: Structural basis of the mechanism and inhibition of a human ceramide synthase
Source: Nat Struct Mol Biol. 2024 Nov 11;32(3):431–40. doi: 10.1038/s41594-024-01414-3 (PMC11919693; doi:10.1038/s41594-024-01414-3)
Supplement: Supplementary file 1 — Supplementary Note 1 and Discussion. [file 41594_2024_1414_MOESM1_ESM.pdf]

nature structural & molecular biology

Supplementary information https://doi.org/10.1038/s41594-024-01414-3

Structural basis of the mechanism and inhibition of a human ceramide synthase

In the format provided by the authors and unedited

Supplementary Note 1

In examining our CerS6 cryo-EM maps, we noted both conditions clearly contain an ordered lipid density near the dimer interface, adjacent to the LH-TM1 loop and TM4. There, we modelled a phosphatidylcholine, as its head group is consistent with the shape, size and environment of the density (Extended Data Fig. 3e). The choline head group is nestled between conserved Trp9 and Trp21 in a classic aromatic box for binding quaternary amines[1]. Adjacent to the choline-binding site is Trp14, which is strictly conserved in CerS2-6. Interestingly, mutation of the corresponding tryptophan residue in CerS3 has been found in patients with ARCI, and the mutant protein was found to be catalytically inactive[2]. In the CerS6 structure, Trp14 does not directly contact the choline head group, but could play a role in forming the lipid-binding site.

Supplementary Discussion: Reaction mechanism of ceramide synthases and entry path of sphingoid bases

Recently, Zelnik et al. postulated that CerS2, and ceramide synthases more generally, use a ternary complex reaction mechanism[3]. In this model, the substrates bind orthogonally, and the long-chain base would access the active site through a side entrance located near the membrane mid-point, where it would bind parallel to the membrane plane. This proposed substrate-binding arrangement is similar to that of small-molecule membrane-bound O-acyltransferases (MBOATs) and led to the suggestion that CerS may catalyze the direct transfer of the acyl chain from acyl-CoA to the long-chain base in a single step involving the two active site histidines[3]. In addition, while this manuscript was under review, the cryo-EM structure of yeast Lac1p bound to an acyl-CoA was reported. That structure revealed a small lateral opening in Lac1p adjacent to the active site[4]. This observation led the authors to

hypothesize that it could potentially correspond to the entry route for sphingoid base substrates[4]. However, this lateral opening is not present in our cryo-EM structures of CerS6 or in our molecular dynamics simulations. Instead, our experimental structures and biochemical studies of CerS6 present several pieces of evidence which support a ping-pong type mechanism, and challenge a ternary complex reaction. First, CerS possess a completely different fold to that of the MBOATs. Indeed, CerS6's structure is strikingly similar to the fatty acid elongase ELOVL7, forming a 6-TM barrel that encloses a narrow tunnel with an essential and structurally superimposable active site histidine pair, which we have also recently shown to catalyze acyl chain transfer via a ping-pong reaction mechanism[5]. Further, purification of a stable acyl-CerS6 adduct species is incompatible with a ternary complex reaction mechanism. Rather, by incubating acyl-CerS6 with sphinganine, FB_1 or FTY720, we show that this species is a *bona fide* reaction intermediate which results in the expected product upon exposure to the second substrate. Additionally, we captured the product-bound state in the N-acyl FB_1-bound structure, where the toxin's hydrocarbon chain binds perpendicularly to the membrane plane and interacts with the hydrophobic face of the central cavity formed by TM5-7. This binding mode in the N-acyl FB_1-bound structure conflicts with a MBOAT-style substrate coordination, but is still consistent with the action of CerS on the fluorescently labelled NBD-sphinganine[6] as the bulky NBD moiety would sit near the wide cytoplasmic entrance of the central cavity. Crucially, FB_1 is a structural analog of sphinganine which undergoes N-acylation similarly to sphingoid base substrates, and its mode of inhibition is competitive towards sphinganine[7], implying that FB_1 and sphinganine likely bind to CerS in a similar manner. In aggregate, our data do not support a ternary complex reaction mechanism. Rather, an ELOVL-type ping-pong model is consistent with both our newly determined structures and biochemical results, and previously reported CerS mutants[8-10].

Supplementary References

1 Dougherty, D. A. The cation-π interaction. *Acc Chem Res* **46**, 885-893 (2013). https://doi.org/10.1021/ar300265y

2 Eckl, K. M. *et al.* Impaired epidermal ceramide synthesis causes autosomal recessive congenital ichthyosis and reveals the importance of ceramide acyl chain length. *J Invest Dermatol* **133**, 2202-2211 (2013). https://doi.org/10.1038/jid.2013.153

3 Zelnik, I. D. *et al.* Computational design and molecular dynamics simulations suggest the mode of substrate binding in ceramide synthases. *Nat Commun* **14**, 2330 (2023). https://doi.org/10.1038/s41467-023-38047-x

4 Xie, T. *et al.* Structure and mechanism of a eukaryotic ceramide synthase complex. *Embo j*, e114889 (2023). https://doi.org/10.15252/embj.2023114889

5 Nie, L. *et al.* The structural basis of fatty acid elongation by the ELOVL elongases. *Nature Structural & Molecular Biology* **28**, 512-520 (2021). https://doi.org/10.1038/s41594-021-00605-6

6 Tidhar, R., Sims, K., Rosenfeld-Gur, E., Shaw, W. & Futerman, A. H. A rapid ceramide synthase activity using NBD-sphinganine and solid phase extraction. *J Lipid Res* **56**, 193-199 (2015). https://doi.org/10.1194/jlr.D052001

7 Merrill, A. H., Jr., van Echten, G., Wang, E. & Sandhoff, K. Fumonisin B1 inhibits sphingosine (sphinganine) N-acyltransferase and de novo sphingolipid biosynthesis in cultured neurons in situ. *J Biol Chem* **268**, 27299-27306 (1993).

8 Spassieva, S. *et al.* Necessary role for the Lag1p motif in (dihydro)ceramide synthase activity. *J Biol Chem* **281**, 33931-33938 (2006). https://doi.org/10.1074/jbc.M608092200

9 Kageyama-Yahara, N. & Riezman, H. Transmembrane topology of ceramide synthase in yeast. *Biochem J* **398**, 585-593 (2006). https://doi.org/10.1042/bj20060697

10 Vanni, N. *et al.* Impairment of ceramide synthesis causes a novel progressive myoclonus epilepsy. *Ann Neurol* **76**, 206-212 (2014). https://doi.org/10.1002/ana.24170
