# Supplementary material for: Structural basis of the mechanism and inhibition of a human ceramide synthase
Source: Nat Struct Mol Biol. 2024 Nov 11;32(3):431–40. doi: 10.1038/s41594-024-01414-3 (PMC11919693; doi:10.1038/s41594-024-01414-3)
Supplement: Supplementary file 5 — Source data of expression. [file 41594_2024_1414_MOESM5_ESM.pdf]

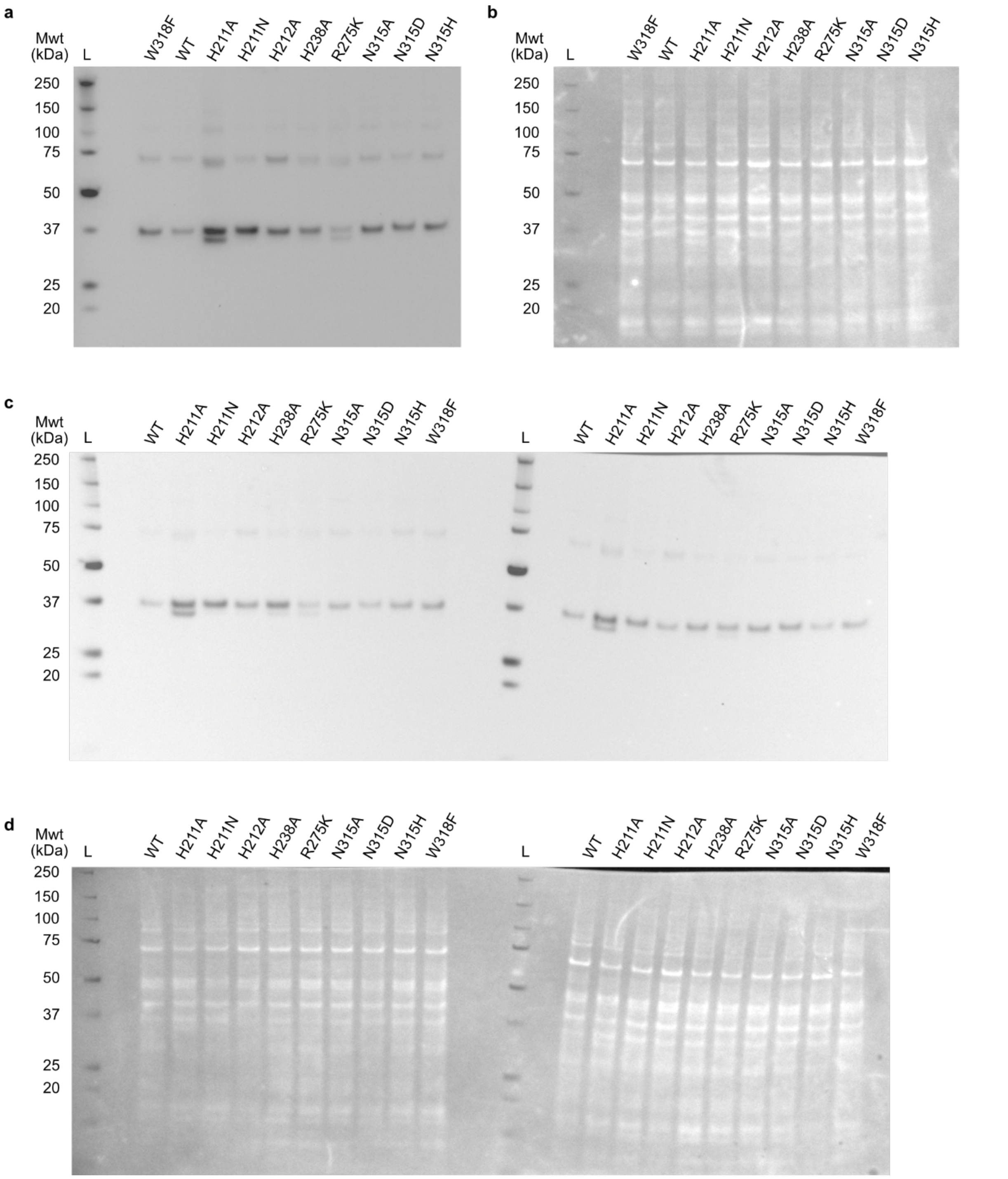

Source Data Fig. 3e. Analysis of CerS6 mutant expression. Western blots of membrane samples used for the activity assay shown in Fig. 3e, complete with SYPRO Ruby total protein staining of the nitrocellulose membrane after the transfer step. **a**, Western blot of biological replicate set 1. **b,** SYPRO Ruby staining of biological replicate set 1. **c,** Western blot of biological replicate sets 2 (left) and 3 (right). **d,** SYPRO Ruby staining of biological replicate sets 2 (left) and 3 (right).
