# Supplementary figures and images for: Structural basis of the mechanism and inhibition of a human ceramide synthase
Source: Nat Struct Mol Biol. 2024 Nov 11;32(3):431–40. doi: 10.1038/s41594-024-01414-3 (PMC11919693; doi:10.1038/s41594-024-01414-3)

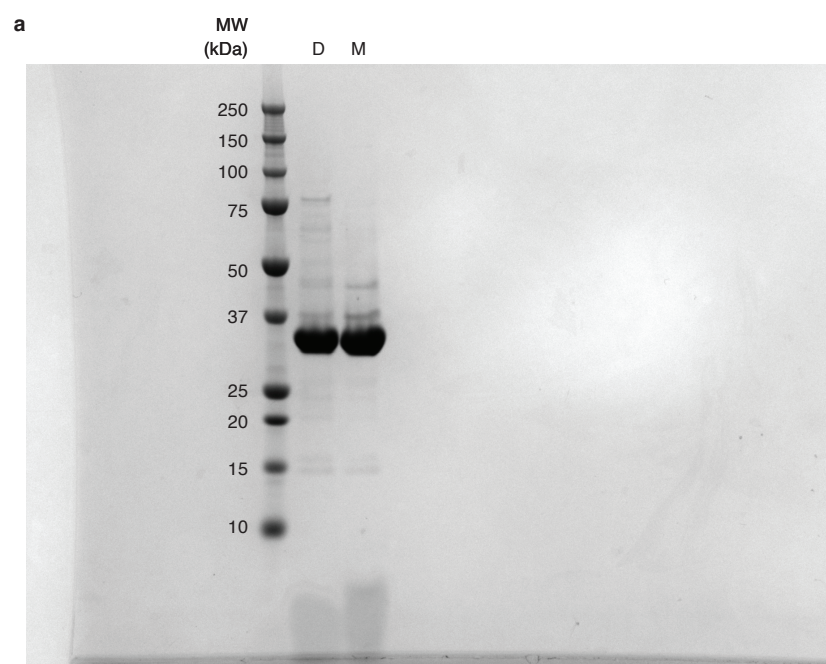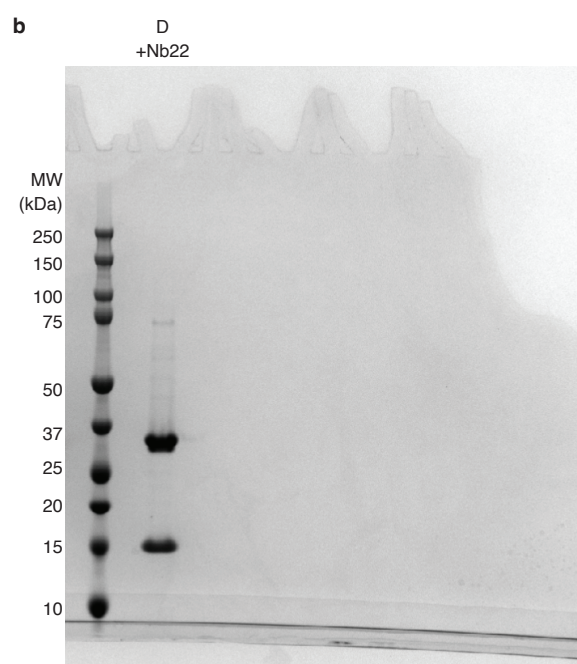

Source data for gels presented in (a) Extended Data Fig. 1b and (b) Extended Data Fig. 1d

Supplement: Supplementary file 8 — Source data of unprocessed gels. [file 41594_2024_1414_MOESM8_ESM.pdf]
